# EBM BLS: Semaglutide Reduces Kidney Disease Progression in Patients with Type 2 Diabetes and Chronic Kidney Disease

**DOI:** 10.1007/s11606-025-09906-8

**Published:** 2025-11-18

**Authors:** Leah S. Alemu, Sweta Narasimhan, Jason T. Alexander

**Affiliations:** 1 Department of Medicine, University of Chicago, 5841 S Maryland Ave, MC 3051, Chicago, IL 60637 USA; 2 Baylor Scott and White Family Medical Center, Waxahachie, TX USA

**Keywords:** chronic kidney disease, semaglutide, type 2 diabetes

Perkovic V, Tuttle KR, Rossing P, et al. Effects of semaglutide on chronic kidney disease in patients with type 2 diabetes. New England Journal of Medicine. 2024 Jul 11;391(2):109–21. 10.1056/nejmoa2403347.

## WHY THIS IS IMPORTANT

- Type 2 diabetes mellitus (T2DM) is an independent risk factor for chronic kidney disease (CKD), end stage renal disease (ESRD), cardiovascular disease, and cardiovascular-related death.^1^
- One third of individuals in the United States with T2DM have CKD.^1^ Despite optimization on guideline directed medical therapies for CKD, many continue to lose kidney function, develop ESRD, and die of primarily cardiovascular complications.
- Glucagon-like peptide-1 (GLP-1) receptor agonists are becoming increasingly utilized in patients with T2DM, and studies have suggested that they may preserve kidney function.^2^

## INTERVENTION

- Participants (n = 3,533) were randomized to receive semaglutide or placebo in a 1:1 ratio.
- Semaglutide dose was escalated over 8 weeks to achieve a goal dose of 1 mg. A lower dose was permitted if dose escalation was not tolerated.
- The primary outcome was onset of kidney failure, sustained reduction of eGFR ≥ 50%, or death from cardiovascular or kidney-related causes.

## RESULTS

**Figure 1:**
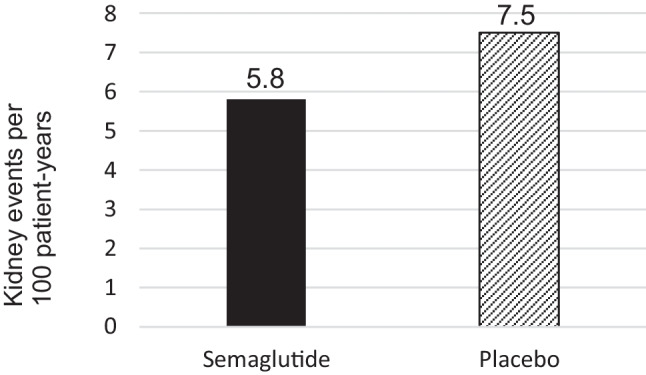
Comparison of kidney disease-related events per 100 patient-years.
- Benefits of semaglutide vs placebo were observed among multiple components of the primary outcome including sustained reduction of eGFR ≥ 50% (9.3% vs 12.1%, HR = 0.73, 95% CI 0.59 to 0.89) and death from cardiovascular causes (12.3% vs 14.7%, HR = 0.71, 95% CI 0.56 to 0.89).
- Secondary outcomes including a composite of death from cardiovascular causes, nonfatal myocardial infarction, and nonfatal stroke (HR = 0.82, 95% CI 0.68 to 0.89), and death from any cause (12.8% vs 15.8%, HR = 0.80, 95% CI 0.67 to 0.95) also favored semaglutide.
- Adverse events leading to treatment discontinuation were higher in the semaglutide group, driven by gastrointestinal disorders (13.2% vs 11.9%).

- Mean age was 66.6 years old, 30.3% were female, 65.8% were White, 23.9% were Asian, and 4.5% were Black. 15.7% were ethnically Hispanic or Latinx.
- At baseline, mean hemoglobin A1c was 7.8%, mean body mass index was 32.0 kg/m2, 68.3% had an estimated glomerular filtration rate (eGFR) between 30 and 60 mL/min/1.73 m2, median urinary albumin-to-creatinine ratio was 567.6, 95.3% were on an angiotensin-converting-enzyme inhibitor or angiotensin-receptor blocker, and 15.6% were on a sodium glucose co-transporter-2 inhibitor.
- The trial was stopped early for benefit. The average length of follow up was 3.4 years.
- The primary composite outcome occurred less frequently in participants receiving semaglutide compared to placebo (hazard ratio (HR) = 0.76, 95% CI 0.66 to 0.88) (Fig 1). Number needed to treat to prevent one primary outcome event over 3 years = 20 (95% CI 14 to 40).

## STUDY DESCRIPTION

### Setting

- Double-blind, randomized, placebo-controlled trial conducted in 28 countries from 2019 to 2021.
- Participants were adults with T2DM with a hemoglobin A1c < 10%, CKD with an eGFR of ≥ 50 to 75 mL/min/m^2^ and a urinary albumin-to-creatinine ratio > 300 and < 5000, or an eGFR 25 to < 50 mL/min/m^2^ and a urinary albumin-to-creatinine ratio > 100 and < 5000, and were on a maximally tolerated angiotensin-converting-enzyme inhibitor or angiotensin-receptor blocker.

### Exclusion Criteria

- Individuals who were pregnant or planning to become pregnant, used a GLP-1 receptor agonist within 30 days of screening, had a recent acute coronary syndrome, were initiating dialysis, had uncontrolled retinopathy or maculopathy, or had New York Heart Association class IV heart failure were excluded.

## STUDY QUALITY AND APPLICATION TO PATIENTS

- The study quality is good.
- Its strengths include the trial size, randomization, and use of clinically meaningful endpoints.
- Limitations include the trial was stopped early for benefit and was sponsored by Novo Nordisk, which also participated in data review and analysis.
- While appropriate statistical methods were employed to control type I error for the early trial termination, benefits observed in the trial are still likely to be inflated.^3^
- Neither sodium-glucose cotransporter-2 inhibitors nor nonsteroidal mineralocorticoid-receptor antagonists were approved for CKD at the time of this trial; whether additional benefit is conferred by adding a GLP-1 receptor agonist to these agents was not evaluated.
- The majority of participants were men (69.7%) and White (65.8%); since CKD has a slightly higher prevalence in women compared to men, and disproportionately affects Black and Indigenous American populations,^1^ the demographics of the trial somewhat limits its generalizability.

## Declarations

### Prior Presentations

This work has not been previously presented at any conferences.
